# Biomarkers and Their Possible Functions in the Intestinal Microenvironment of Chagasic Megacolon: An Overview of the (Neuro)inflammatory Process

**DOI:** 10.1155/2021/6668739

**Published:** 2021-04-07

**Authors:** José Rodrigues do Carmo Neto, Yarlla Loyane Lira Braga, Arthur Wilson Florêncio da Costa, Fernanda Hélia Lucio, Thais Cardoso do Nascimento, Marlene Antônia dos Reis, Mara Rubia Nunes Celes, Flávia Aparecida de Oliveira, Juliana Reis Machado, Marcos Vinícius da Silva

**Affiliations:** ^1^Department of Bioscience and Technology, Institute of Tropical Pathology and Public Health, Federal University of Goiás, Goiânia, GO, Brazil; ^2^Department of General Pathology, Federal University of Triângulo Mineiro, Uberaba, Minas Gerais, Brazil; ^3^Department of Microbiology, Immunology and Parasitology, Institute of Biological and Natural Sciences, Federal University of Triângulo Mineiro, Uberaba, Minas Gerais, Brazil

## Abstract

The association between inflammatory processes and intestinal neuronal destruction during the progression of Chagasic megacolon is well established. However, many other components play essential roles, both in the long-term progression and control of the clinical status of patients infected with *Trypanosoma cruzi*. Components such as neuronal subpopulations, enteric glial cells, mast cells and their proteases, and homeostasis-related proteins from several organic systems (serotonin and galectins) are differentially involved in the progression of Chagasic megacolon. This review is aimed at revealing the characteristics of the intestinal microenvironment found in Chagasic megacolon by using different types of already used biomarkers. Information regarding these components may provide new therapeutic alternatives and improve the understanding of the association between *T. cruzi* infection and immune, endocrine, and neurological system changes.

## 1. Introduction

According to the World Health Organization (WHO), approximately 8 million people in the world are infected with *Trypanosoma cruzi* (*T. cruzi*), the etiological agent of Chagas disease (CD) (WHO, 2018). Most of these cases are concentrated in Latin America, where the number of new cases per year is estimated to be 300,000 [1]. In Brazil, at least one million people are infected with *T. cruzi*, and most of them are in the chronic phase [2]. Recently, the cases of acute CD have increased in Brazil, mainly owing to the consumption of food contaminated with parasites, such as açai and sugarcane juice [3, 4].

CD has two phases, acute and chronic. The acute phase is characterized by the high parasitic load with cellular destruction and extensive inflammatory foci [5]. Subsequently, the condition may progress to an undetermined or determined chronic phase of the disease, which may then lead to the development of severe cardiac and digestive dysfunctions and death [5]. The indeterminate chronic phase is characterized by positive serology and/or parasitology along with the absence of symptoms and/or signs caused by *T. cruzi* [5]. The symptomatic chronic phase can develop after few years to decades due to some unknown factors, in approximately 30% of the infected people. In this phase, mega syndromes could develop with cardiac (cardiomegaly) and/or digestive (megaesophagus and/or megacolon) involvement, in addition to neurological damage [5].

The first suspected case of the digestive form of CD appeared in 1916, when Carlos Chagas observed that in the acute infection some adults presented marked dysphagia, which was perpetuated in the chronic phase, and he called this “Choking Disease” [6]. Patients with the digestive form of CD present with hypertrophic changes with esophagus (megaesophagus) and/or intestinal colon (megacolon) dilation, leading to dysphagia, regurgitation, and motor discoordination. The main cause of dilation of these organs is their denervation owing to the destruction of myenteric or Auerbach and submucosal or Meissner plexus [7]. Different mechanisms have been proposed to explain this neuronal destruction, such as the release of toxins during parasite fragmentation, direct neuronal injury through cell parasitism, and inflammatory damage triggered by the parasite [8].

This review focuses on the intestinal biomarkers of Chagasic megacolon and their possible functions in CD progression, especially those related to (neuro) inflammatory processes. Despite the advances in the understanding of the CD evolution, cell interactions and molecular pathways involved in megacolon development/progression are not completely clear. Thus, the investigation of biomarkers of altered intestinal structures is also fundamental to understand the rich microenvironment of Chagasic megacolon and its relationship with disease pathogenesis, especially those related to neuronal death.

## 2. An Immunological Overview of Chagasic Megacolon

Chagasic megacolon is characterized by a hollow viscera with chronic constipation, followed by permanent pathological dilation of the organ wall, which usually occurs in the rectus sigmoid portion, where the parasite is commonly found [5]. From the morphological point of view, patients with megacolon present histological changes such as organ wall thickening due to severe muscle fiber hypertrophy, especially in the circular layer; these changes are usually associated with an inflammatory process in addition to the presence of hypertrophied neurons in the submucous and myenteric plexus and/or with the signs of degeneration [7].

The myenteric plexus is severely affected by the inflammatory process triggered by *T. cruzi* infection, resulting in decreased number of nerve cells or even complete destruction of nerve ganglia [7, 9]. The *T. cruzi*-induced intestinal inflammation led to neuron degeneration in the gastrointestinal system [10, 11] and ganglionitis, peri-ganglionitis, neuritis, and peri-neuritis in the myenteric plexus [7]. These lesions begin to form in the acute phase and are prolonged and aggravated during the chronic phase [12]. The inflammatory process related to this neuron degeneration usually has a focal distribution. However, some neurons close to the injured neurons remain intact [7].

The phenotypic characterization of the inflammatory infiltrate components led to a better understanding of the pathophysiology of Chagasic megacolon, although the process is not yet completely understood. Eosinophils, mast cells (MCs) and their proteases, CD68^+^ macrophages, CD57^+^ natural killer cells, and TIA-1^+^ cytotoxic lymphocytes in the affected intestine are factors related to inflammatory process maintenance and neuronal death during the CD chronic phase [8, 13–15].

Over the years, some theories attempted to explain neuronal destruction in the different phases of infection. During the acute phase, the high concentration of the protozoan has been suggested to participate in neuronal death [8]. In contrast, in the chronic phase, in which minimal amount of *T. cruzi* is clearly demonstrated in the injured organ, neuronal destruction process would be the consequence of the (auto) immune response following infection [8]. According to this idea, as indeterminate form progresses to digestive form, peripheral blood mononuclear produces higher levels of IFN-*γ*, inducing TNF-*α* augmentation and a proinflammatory milieu in the intestine [16].

Cytokines such as TNF-*α* and IFN-*γ* (produced mainly by Th1 lymphocytes) at the onset of infection are essential for parasites control as they are involved in the activation of microbicidal macrophages which produce reactive oxygen species (ROS) or nitric oxide (NO) [17, 18]. However, a balanced response is essential so that the infection is controlled and does not result in excessive tissue damage.

In addition to lymphocytes, central or peripheral glial cells are potential sources of NO. They have been reported to participate in neuropathologies, leading to the production of neuron protective or toxic molecules [19, 20]. In Chagasic megacolon, the alteration of the number of enteric glial cells, as well as glial activation markers, suggests that these cells participate in the inflammatory process and in the subsequent neuronal damage or protection [15, 21]. Actually, glial cells have been shown to be parasitized by *T. cruzi* in both the central nervous system (CNS) and the enteric nervous system (ENS) [7, 22–25].

## 3. Biomarkers in Chagasic Megacolon: Immune Response and Neuroinflammation

In addition to glial cells and traditional immune system components, other components are also associated with the progression of or protection against Chagasic megacolon. Possible interactions between the nervous and immune systems that may be associated with different CD progression events, particularly those related to the maintenance of the inflammatory process and tissue damage, especially in the ENS, are discussed below.

### 3.1. Protein Gene Product

Initially described in 1981, protein gene product (PGP 9.5) is a cytoplasmic protein produced by central and peripheral neurons [26]. It is highly concentrated in intact neurons and can be used as a specific neuronal marker and, consequently, for neuronal density evaluation [27]. The use of PGP 9.5 neuronal marker confirmed the participation of the ENS in the pathophysiology of diseases related to neuronal damage, such as Hirschsprung's disease (HD) [28], ulcerative colitis [29], and intestinal neuronal dysplasia [30].

Due to the difficulty to establish experimental models for the digestive CD, divergent results regarding PGP 9.5 have been published. The C3H/He mice, 30 days after infection (acute phase) with Brazil *T. cruzi* strain, did not reduce the intestinal neuronal PGP 9.5 staining as compared with control mice [31]. Similarly, C57Bl/6 mice, seven days after infection (acute phase), also showed no decrease of immunoreactive neurons for PGP 9.5 (IR-PGP 9.5) [32]. In contrast, C57Bl/6 mice demonstrated a reduction in the neuron fibers and IR-PGP 9.5 neurons in the intestine, especially in the myenteric plexus, 10 days (acute phase) after infection with the Y strain [22].

Additionally, PGP 9.5 neuronal density was also investigated in chronic models of CD [33]. Infection of Swiss mice with Y strain for 11 days (acute phase) and 15 months (chronic phase, with the administration of a single dose of benznidazole at 11 days of infection) found that the infection reduced the density and number of IR-PGP 9.5 neurons in the intestine. Furthermore, the number of intramuscular IR-PGP 9.5 neuron fibers was lower in the chronic phase as compared to the acute phase [33].

Although most of the studies on PGP 9.5 neuronal density use the experimental models, da Silveira et al. [15] and Martins et al. [34] showed that patients chronically infected with megacolon presented reduced density of IR-PGP 9.5 nerve fibers in both, internal muscle layer and intestinal external muscle, as compared to nonmegacolon CD patients and noninfected.

### 3.2. Peripherin

Peripherin is a type III intermediate filament protein expressed in the cell body and axons of neurons, mainly in the peripheral nervous system [35–38]. Peripherin may play an important role in diseases such as amyotrophic lateral sclerosis [39–41] and type I diabetes mellitus [42–44]. In addition, peripherin plays a promising role as a pan-neural marker in the intestinal mucosa and submucosa [45].

Peripherin helps in the diagnosis of HD, as it is a part of the highly sensitive and specific ganglion cell identification protocol. Peripherin as neuronal biomarker is more sensitive and superior to microtubule-associated protein-2 (MAP-2) and calretinin in ganglion cell and nerve fibrillation marking in colon and rectum biopsies [46] so is specific method for identify HD patients [47].

The role of peripherin in CD, as well as in other pathological processes, is not completely elucidated, and despite the controversies, this protein has been used as a standard for pan-neuronal staining in intestinal diseases [45, 48, 49]. Morphometric analysis of immunoreactive neuronal ganglia for peripherin in the colon from CD patients with megacolon showed reduction in this protein compared to that in healthy people, as it is related to decreased number of neurons. In addition, neuronal ganglia marked with this protein in CD patients showed a deformed structure [50].

In addition, peripherin seems to play a role in neuronal apoptosis through the interaction with protein kinase C [51]. Considering that one of the hypotheses for the development of CD megacolon is the cell death of mucosal neurons, peripherin may have an additional role in the pathogenesis of this disease.

### 3.3. HuC/HuDa

HuC and HuDa are RNA-binding proteins that are obtained by the alternative splicing of Hu RNA [52], exclusively expressed in neurons. These proteins are considered potent ENS neuron markers [53–55]. Anti-HuC/HuDa antibodies were used as neuronal markers to determine the total number of cell bodies in the nerve plexus of CD patients, pointing that the dilated portion of CD patients with megacolon had fewer neuronal bodies in each ganglion [56]. Similarly, HuC/HuDa were also used as neuronal markers in the colon samples of CD patients in another study [57].

Thus, PGP 9.5, peripherin, and HuC/HuDa were used to evaluate intestinal neuronal loss, especially in Chagasic megacolon, in addition to evaluation of neuronal density, providing a better picture on neuronal regeneration, subpopulation proportion, and cell colocation [58]. Furthermore, they represent an alternative to basic histological staining techniques, which are, in general, nonspecific [58].

### 3.4. Substance P

Substance P (SP) is a neuropeptide belonging to the tachykinin family [59, 60]. Expressed by several cell types, mainly neurons [61], this neuropeptide is involved in important inflammatory mechanisms, either in cell migration, where it acts directly, or through the induction of a series of chemokines, receptors and adhesion molecules in lymphocyte proliferation, and innate and adaptive immunity cell activation [62].

SP acts via the interaction with cell surface receptors NK1R, NK2, and NK3. NK1R has the greatest affinity for SP, as it is predominantly related to inflammatory processes, whereas NK2 and NK3 receptors are more related to gastrointestinal motility responses [63, 64]. In the intestine, SP regulates smooth muscle contractility, epithelial ion transport, vascular permeability, and gastrointestinal tract immune function [65, 66]. NK1R receptor density was found to increase significantly in patients with Crohn's disease and ulcerative colitis [67]. These levels correlate with a poor prognosis of CD [68–70].

Substance P can cause neuroinflammation, since neurons also produce and respond to SP [66]. Classical neuroinflammatory responses are characterized by glial activation, microglial proliferation, leukocyte recruitment, and positive inflammatory mediator regulation and secretion [71]. In infectious diseases, SP increases the severity of inflammation associated with *Trypanosoma brucei* infection, etiological agent of sleeping sickness, for example, and treatment with an NK1R antagonist reduced this effect [72]. Similarly, this neuropeptide also contributed to the development of *Taenia crassiceps* infection in a neurocysticercosis model. Knockout mice for the SP precursor or its NK1 receptor had reduced granuloma volume and lower IL-1*β*, TNF-*α*, IL-6, and IFN-*γ* expression [73]. NKR1 inhibition was also promising for the treatment of experimental autoimmune encephalomyelitis (EAE) [74], head trauma [75], and meningitis caused by *S. pneumoniae* [76]. NKR1 inhibition is a promising treatment for neuroinfectious and neuroinflammatory processes, with positive immune response effects in the nervous tissue, and several antagonists are being developed.

The SP expression has also been shown to be directly correlated with the severity of Chagasic megacolon. The SP expression has been shown to be high in the submucosa and myenteric plexus neurons in the dilated portion of Chagasic megacolon compared to that in the nondilated portions and noninfected people [56]. Moreover, in contrast to the high SP level, low NK1R receptor levels were observed in the dilated portion [77]. Conversely, other studies described lower myenteric and submucous plexus SP concentration in the rectum samples from CD patients. The authors associated this finding with the destruction of intestinal nerve ganglia [78]. Corroborating this study, SP staining activity was reduced in the neurons of the myenteric plexus of the colon in experimental models of acute and chronic infection with the Y [79, 80].

In general, the role of SP in the immune response to *T. cruzi* infection is particularly remarkable. The increase of this neuropeptide in patients with megacolon may be related to the elimination of the parasite, which somehow accelerates the progression of the megaesophagus or colon due to increased inflammatory response. Besides, SP may have the ability to modulate the production of a wide range of cytokines. SP stimulates the production of proinflammatory cytokines such as IL-1*β*, IL-6, and TNF-*α* by human peripheral mononuclear cells, inducing lymphocyte proliferation and immunoglobulin production [62, 65, 81]. This situation probably occurs due to positive NK1R regulation, since, while IL-12, IL-18, and TNF-*α* induce the NK1R expression in T cells [82]; IL-10 and TGF-*β* decrease the NK1R expression [83]. The release of SP-induced inflammatory mediators potentiates tissue injury and stimulates leukocyte recruitment, amplifying the inflammatory response [84]. Thus, these studies suggest that the SP/NK1R axis maintains the inflammatory response to *T. cruzi* infection in CD, but no study has analyzed the effects of SP on intestinal Chagas neuroinflammation.

### 3.5. Growth-Associated Protein 43

Growth-associated protein-43 (GAP-43) is a marker of neuronal plasticity in embryogenesis processes, axonal growth and regeneration, and subsequent neurite branching [85]. GAP-43 has already been used in several intestinal disease models to evaluate neuronal plasticity, such as HD [86, 87], inflammatory bowel disease (IBD) [88], appendicitis [89], intestinal neuronal dysplasia [90], and *Nippostrongylus brasiliensis* infection [91]. These conditions are characterized by neuronal loss and establish an intestinal inflammatory process, which are also essential factors for the progression of Chagasic megacolon.

In fact, evidence shows that the neuronal regenerative process, evaluated using GAP-43 expression, occurs in the intestinal nervous plexus in Chagasic megacolon [50, 92]. Neuronal destruction mediated by the inflammatory process in the most affected areas is suggested to induce neurons to extend their projections into the destroyed areas to maintain intestinal homeostasis [50, 92]. However, this regeneration would be restricted only to the most destroyed neural subpopulations in the intestinal form of CD, corresponding to inhibitory motor neurons characterized by the expression of intestinal vasoactive peptide and NO synthase [50]. The same reestablishment would not occur for other subpopulations such as intrinsic primary afferent neurons, excitatory motor neurons, and interneurons since they are not considerably destroyed by the infectious process [50].

Although no studies have focused on GAP-43-related pathways in Chagasic megacolon, other axon injury models have been evaluated [93–96]. Some studies have shown that GAP-43 inhibition decreases axon regenerative capacity after injury [93]. In addition, the use of blockers to inhibit the GAP-43 process, such as Nogo-A-dependent processes, represents an alternative to induce increased axon regeneration [97–99]. Thus, studies on neuronal regeneration and plasticity in Chagasic megacolon may establish new mechanisms to control progression in the early stages of this form of CD.

### 3.6. S-100 Proteins and Glial Fibrillary Acidic Protein: Enteric Glial Cells

S-100 is a family of approximately 21 proteins characterized as small calcium-binding proteins [100]. Besides participating in Ca_2_^+^ homeostasis, S-100 proteins have intracellular and extracellular functions, acting in an autocrine and/or paracrine manner in target cells [101] and act in cell proliferation, differentiation, and migration as well as inflammatory processes [102], and as cell markers, mainly of glial cells [103].

Although S-100 proteins are mainly used as a CNS astrocyte biomarker [104, 105], glial components of the ENS also showed reactivity for these proteins, which was not observed for intestinal neurons [106, 107]. Thus, S-100 was characterized as a pan-glial marker also for the intestine.

Another important marker to visualize glial components, specifically astrocytes, is the glial fibrillary acidic protein (GFAP) [108]. Classified as the main intermediate filament protein of class III astrocytes, GFAP is related to the maintenance of cell structure and signal transduction [109]. In general, GFAP has been shown to play a role in cell motility, migration, and proliferation processes; chaperone-mediated autophagy; synaptic plasticity; neuronal damage or protection; and inflammation [108, 110]. Initially described as a CNS astrocyte marker, astrocyte-like enteric glial cells were also found to be immunoreactive for GFAP, especially in the intestinal nerve ganglia [103, 104, 111].

Chagasic megacolon was characterized by decreased S-100 glial-IR cells in different intestinal nerve plexuses [15, 112, 113]. In addition, the dilated portion of the intestinal form of CD is described as having greater glial destruction (IR-S-100) than the nondilated one [15, 21, 114]. Interestingly, the GFAP marker increased the number of glial cell immunoreactive for this protein in *T. cruzi* infection, regardless of intestinal involvement [15, 21]. However, the number of IR-GFAP glial cells in CD patients without megacolon or in the nondilated part of the intestine of CD patients was higher than that in the affected part [15, 21]. Thus, considering that unaffected intestinal segments would have greater glial cell preservation, the presence of these cells has been suggested to protect ENS components from the inflammatory process established by *T. cruzi* infection [15, 21].

In fact, enteric glial cells and their products participate in intestinal homeostasis, especially in neuronal survival. Thus, the reduction of these cells has already been suggested to be associated with infectious [115, 116] and noninfectious intestinal diseases [117–119], which are characterized by neurodegeneration, like in CD. Evidence shows a glial cell and neuron network owing to the production of NTs such as NGF, NT-3/4/5, and glial cell line-derived neurotrophic factor (GDNF). These neurotrophic factors directly and positively affect neuronal growth, maturation, and survival [120, 121]. Whether these molecules could protect neurons was also evaluated in the digestive form of CD [112]. Interestingly, the levels of NT-3, NGF, and GDNF were higher in the intestine of CD patients without megacolon, and this increase was correlated with the increase in the number of IR-GFAP glial cells and ENS cell protection [112]. In addition, along with the glial system, neurons and inflammatory infiltrate also increased NT production, increasing the relationship between the immune and nervous systems [112]. Thus, the authors suggested that the inflammation found in the intestine of CD patients without megacolon is determinant for NT production and that the increased enteric glial cells would facilitate the reestablishment of the integrity and functioning of the intestine after *T. cruzi* infection and the regulation of the established inflammatory process.

Similar to CNS astrocytes, enteric glial cells are sensitive to the stimuli of inflammatory processes. Proinflammatory cytokines such as IL-1*β*, TNF-*α*, and IFN-*α* can induce GFAP [122, 123] and NT production [124] and act on *in vitro* proliferation of these cells, which could justify the results found in the intestine of patients infected with *T. cruzi*. Additionally, experimental models of intestinal inflammation showed that inflammation induces enteric glial cell proliferation in the myenteric plexus and that the lack of these cells results in severe tissue inflammation and intestinal necrosis [125, 126]. Conversely, enteric glial cells (ECGs) can also produce inflammatory mediators and exacerbate the intestinal inflammatory process [122, 127–129].

In fact, the microglia phenotype in the CNS is directly related to the microenvironment to which these cells are exposed [130]. When these cells are stimulated with proinflammatory components such as IFN-*γ*, TNF-*α*, and IL-17, the microglia polarize to the M1-like profile and can produce NO, ROS, and proinflammatory cytokines (IL-1, IL-12, and TNF-*α*) related to neurotoxicity and neuronal death. In contrast, once IL-4 and IL-13 stimulate these cells, the M2-like profile is established in the microglia. This activation profile is related to IL-10, TGF-*β*, and NT production (NT-3, NGF, and GDNF), which is related to neuroprotection and neuronal survival. Although this classification was not noted for EGCs, evaluating intestinal subpopulations and glial plasticity in CD, especially comparing infected patients with and without megacolon, is important.

In addition, analysis of neurodegeneration and inflammatory processes showed that CNS microglia cells express molecules originally found in macrophages, such as class II HLA-DR, B7.1, and B7.2, indicating how these cells interact with the immune system as antigen-presenting cells. In fact, in CD patients with megacolon, EGCs begin to express class II HLA-DR and B7 costimulating molecules [49]. These results suggest that the established inflammatory process would affect the profile of EGCs that could act on lymphocyte activation in the intestine of patients with megacolon [49]. The presence of macrophages and lymphocytes was often associated with the severity of Chagasic megacolon [13]. Thus, EGCs also influence the progression of chronic digestive CD as antigen-presenting cells, consequently acting on lymphocyte activation [49].

**Figure 1:**
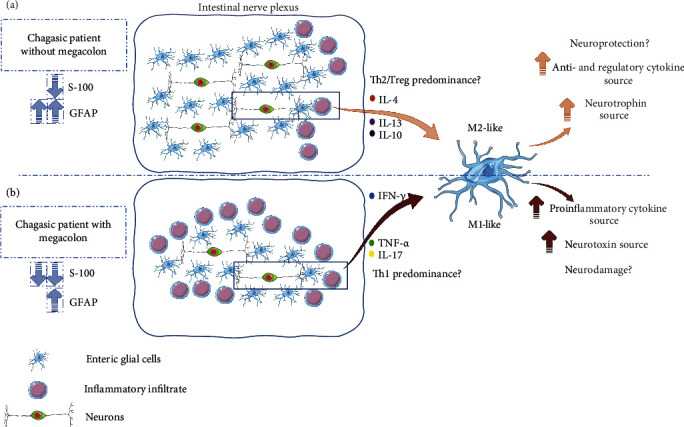
Hypothetical scheme of the differential behavior of enteric glial cells in the intestine of infected patients without megacolon (a) and with megacolon (b). This work, “Hypothetical scheme of the differential behavior of enteric glial cells in the intestine of infected patients without megacolon (a) and with megacolon (b),” is a derivative of “Servier Medical Art” by Servier, used under CC BY 3.0.

These results suggest that the progression of intestinal CD depends on the microenvironment of the intestinal segment (with or without megacolon; Figure 1). The presence of a greater inflammatory infiltrate, a higher level of proinflammatory cytokines, and tissue destruction may indicate that EGCs would maintain the inflammatory process and neuronal destruction in Chagasic megacolon. The opposite could be assumed for patients without the digestive form, with lower organ involvement and greater quantity of enteric glia reestablishing the intestinal homeostasis. Further studies are needed to determine the role of EGCs in the acute phase of the disease and to elucidate whether this phase would be the determinant for the activation, destruction, and subsequent participation of the glia in the development of Chagasic megacolon, as well as whether the activation profile of these cells in the intestine of *T. cruzi-*infected patients in the chronic phase is M1- or M2-like.

### 3.7. Tryptase and Chymase: Mast Cells

Tryptase and chymase are the main serine proteases secreted by MCs; they mainly act as a factor to evaluate MC activation and classification in humans and mice [131, 132]. In general, tryptase and chymase are mainly involved in extracellular matrix degradation processes, tissue remodeling, and fibrosis [133–136] and have a dualistic role in inflammatory processes [137].

In fact, MCs play an important role in megacolon pathophysiology. Evidence shows that the concentration of these cells is increased regardless of the affected intestine layer [13, 34, 138, 139]; the same has been observed in a murine chronic phase model [140]. The increase in chymase-IR and tryptase-IR MCs has been shown to be correlated with decreased PGP 9.5-IR neurons in Chagasic megacolon [34]. In addition, only increased tryptase-IR MCs (and not chymase-IR MCs) were correlated with decreased PAR2-IR neurons. This receptor is cleaved by tryptase and participates in hyperactivation and neuronal death, triggering chronic intestinal function changes [141]. Also, there is a proximity between MCs in active degranulation (fragmented and anaphylactic) and intestinal nerve fibers, and the subsequent neuronal death could be partially mediated by the tryptase enzyme released through MC degranulation in Chagasic megacolon, and both proteases would be involved in the maintenance of the inflammatory process in the organ [34].

The communication between MCs and neurons in the intestine has already been reported in other studies [141–144], suggesting an important interface between the immune and nervous systems [145]. MC degranulation was found to be associated with nerve fiber network disintegration and loss of neuronal cell bodies in a coculture model between myenteric neurons and peritoneal MCs isolated from rats [146], and besides tryptase, other proinflammatory MC mediators such as IL-6 and prostaglandin 2 could induce neuronal death. The same was observed when a PAR2 agonist was used [146], corroborating what was suggested by Martins et al. [34] in Chagasic megacolon.

Neuropeptides have also been shown to activate MCs, which further confirms the participation of this cellular type in nervous system inflammation [147], such as SP in experimental dermatitis model [148] and irritable bowel syndrome [149]. Thus, the increased SP-IR neurons found in Chagasic megacolon are thought to be correlated with MC degranulation in the affected organ, subsequently releasing proinflammatory mediators such as TNF-*α*, IL-1*β*, and tryptase, which reinforced the relation between MCs, tryptase, neuroinflammation, and neuronal death.

Moreover, tryptase was suggested to participate in the inflammatory cell transmigration process [150–153]. Lung allergy [154, 155], psoriasis [156], asthma [157], and CD models [158] showed that this protease is a potential component in eosinophilic infiltration. The increased tryptase-IR MCs in the myenteric plexus and internal muscle layer were positively correlated with increased eosinophils in the same regions in Chagasic megacolon [158] and showed cell proximity under electron microscopy, suggesting a possible communication between MCs and eosinophils via PAR2. The role of MCs–eosinophil communication during the *T. cruzi* infection is unknown, but MCs are believed to participate in eosinophil survival in Chagasic megacolon [158]. However, MCs–eosinophil cells interact through the production of mediators such as histamine, eotaxin, and tryptase produced by MCs [159] and via physical contact in other intestinal diseases [160]. A decreased eosinophil infiltration was also observed under MC deficiency conditions [161], which reinforces the communication between these two cell types.

The presence of eosinophils in the *in vitro* and/or *in vivo* CD study models in humans was associated with protozoan destruction and control, tissue damage, and inflammatory process maintenance in both the acute and chronic phases of the disease [162–168]. In addition, this cell type was correlated with intestinal fibrosis in the nerve plexus and muscle layers of patients with megacolon, regions that showed an increased level of eosinophils compared to that in noninfected people and infected patients without megacolon [13]. The dual role of these cells in the affected intestine has been discussed. Increased eosinophils would allow (1) returning to intestinal homeostasis or/and (2) controlling the infection and consequently resulting in tissue damage.

The role of chymase in the inflammatory process is not yet clear. Evidence suggests that it participates in the pathophysiology of intestinal inflammatory diseases such as IBD [169] and protozoan [170] and helminth infections [171]. Therefore, the blockade of this protease is related to an improved intestinal inflammatory status [172, 173], which was related to increased IL-10, TGF-*β*, IL-17A, and T regulatory lymphocyte production in experimental IBD in rats [174].

In contrast, the expression of mast cell chymase gene (CMA1) was shown to be associated with increased IL-10 in an experimental chronic intestinal stress model [175], suggesting its regulatory role in the inflammatory process. In addition, a murine sepsis model showed that the mast cell protease 4 (corresponding to chymase in humans) degrades TNF-*α*, limiting the proinflammatory effect of this cytokine, which increased the survival of the animals [176]. The functions of these proteases are apparently dependent on the location of MCs producing it, which also varies according to the pathophysiology of each disease studied.

Besides the inflammatory process, chymase participates in tissue remodeling via TGF-*β* and metalloproteinases in different organs and pathological conditions [177, 178]. The presence of intestinal MCs in Chagasic megacolon is positively correlated with organ fibrosis [13]. Thus, increased chymase in the chronic phase of CD may represent a pathway for intestinal collagen deposition according to disease progression. However, little is known about this function in Chagasic megacolon.

**Figure 2:**
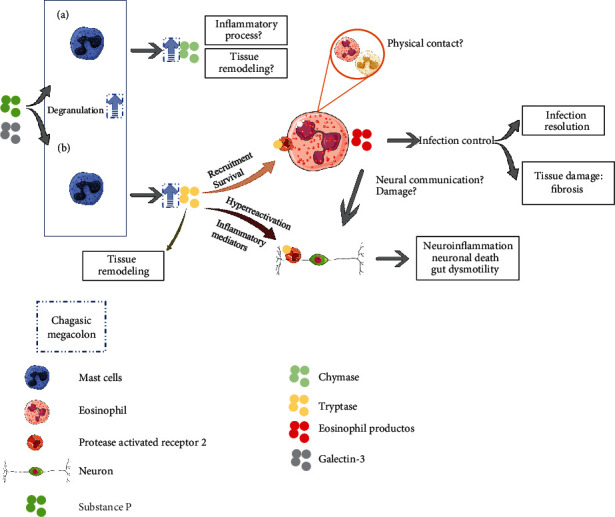
Possible functions of mast cells and chymase (a) and tryptase (b) proteases in Chagasic megacolon. This work, “Possible functions of mast cells and chymase (a) and tryptase (b) proteases in Chagasic megacolon,” is a derivative of “Servier Medical Art” by Servier, used under CC BY 3.0.

Proteases produced by enteric MCs represent a source of therapeutic targets for Chagasic megacolon. Thus, further studies are needed to elucidate more completely the mechanisms involving these cells and their products since they can be related to several processes that affect the progression of Chagasic megacolon (Figure 2).

### 3.8. Galectins

Galectins are proteins of the lectin family that have high affinity for the *β*-galactoside residues present in the components of the extracellular matrix (ECM). Although all cells express galectins, the amount expressed varies according to cell type, tissue, and microenvironment. Approximately 15 types of galectins have been reported in vertebrates, with different body functions [179]. In general, these proteins are related to homeostasis conditions, inflammatory processes, and interactions with ECM components and between cells, cell adhesion, and fibrogenesis.

Changes in galectin levels were correlated with the progression and worse prognosis of intestinal diseases such as colorectal cancer, colorectal adenoma, rectal cancer, and Crohn's disease [180]. In CD, galectin-1 (gal-1) and galectin-3 (gal-3) production was analyzed in experimental *in vitro* cardiac form models and in infected patients with Chagas heart disease in the acute and chronic phases [181]. Information regarding the profile and function of these proteins in the megacolon is lacking.

Beghini et al. [182] were the first to show changed gal-1, gal-3, and gal-9 levels in the intestine of *T. cruzi*-infected patients with megacolon. The authors reported that these three galectins were highly expressed only in the myenteric plexus of patients with the digestive form of CD compared to those who were not infected. In addition, to better establish the gal-3 profile in Chagasic megacolon, Garvil et al. [183] analyzed the protein expression in the intestine of megacolon patients with intact or injured mucosa and in people without infection with an intact mucosa. Regardless of mucosal integrity, patients with Chagasic megacolon had more gal-3-marked cells [183]. Both studies suggested the potential functions of galectin in Chagasic megacolon.

Studies have shown that gal-1 has an ambiguous role in the course of *T. cruzi* infection, either acting in a protective or detrimental manner. An *in vitro* cardiomyocyte infection model showed that the use of endogenous gal-1 inhibited *T. cruzi* infection [184]. The same study showed that gal-1–deficient mice had higher parasitemia, less tissue inflammation, and lower survival than wild mice, suggesting an important role of gal-1 in protection against infection [184]. In contrast, another study showed that this galectin can induce anergy and form dendritic and T regulatory tolerogenic lymphocyte cells in mice with acute *T. cruzi* infection. This would impair Th1 response, which is important for controlling infection, thereby increasing parasite persistence in the tissues [185].

Besides its impact on the course of infection, gal-1 was also found to be related to neuroprotection in neuroinflammatory diseases [186]. Thus, the increase of this galectin in Chagasic megacolon could be to protect the ENS and control the neuroinflammatory process. In a murine multiple sclerosis model knockout for gal-1, extracellular gal-1 and also gal-9 could induce Th1 and Th17 lymphocyte apoptosis, which decreased the established inflammatory process [187, 188]. In addition, the administration of endogenous gal-1 in this same wild murine model inhibited microglia activation for the M1-like profile (important in EAE pathophysiology), favoring M2-like polarization and neuroprotection in this pathology [189]. Gal-1 also induces macrophages to produce NTs, stimulates the regenerative process of axons, and facilitates Schwann cell migration to injured peripheral nerves [190]. Thus, future studies need to elucidate the role of gal-1 in the inflammatory process in the chronic phase of CD and its relationship with ENS components.

Considering that Gal-3 level increases in the myenteric plexus of CD patients with megacolon, its role in facilitating the establishment of cell invasion at the early stages of infection, maintenance of the inflammatory process, and induction of fibrosis in the intestine has been discussed [182, 183]. In fact, these processes were already revealed in experimental Chagas cardiopathy models, in which the increase of this lectin was found to be directly related to increased inflammatory processes and cardiac fibrosis. Gal-3 also has a relationship with MCs by inducing their degranulation, a process involved in collagen fiber formation and tissue remodeling by tryptase [191]. Thus, gal-3 could have a detrimental role in the Chagasic megacolon and could be involved in the progression of the digestive form in both the persistence of the inflammatory process and the induction of tissue remodeling.

Gal-3 also plays an essential role in neuroinflammation [186], a process that may represent a new pathway for the maintenance of the neural inflammatory process in Chagasic megacolon. An experimental EAE model with a knockout phenotype for gal-3 suggested that this lectin would exacerbate the inflammatory process of the disease by increasing IL-17 and IFN-*γ* and decreasing IL-10 [192]. As for the ENS, a murine stroke model (middle cerebral artery occlusion) showed that serum from C57Bl/6 mice overexpressing gal-3 decreased the *in vitro* survival of intestinal myenteric neurons [193]. The same was observed when these cells were exposed to purified gal-3 [193]. Finally, the authors concluded that gal-3 release in the central and peripheral nervous systems would be directly related to intestinal neuronal death via toll-like receptor 4, an innate immunity receptor previously related to death induction in neurons [193]. Future studies need to focus on identifying gal-3 inhibitors for the control and treatment of CNS neuroinflammatory pathologies [194]. Thus, the use and search for these inhibitors may also represent alternatives for ENS-related diseases such as Chagasic megacolon.

Further studies are needed to elucidate the role of galectins in the digestive form of CD, as most of the studies available are related to cardiac changes. In general, these lectins may also mediate the interaction between the immune and nervous systems, suggesting new therapeutic targets based on gal-3 blockade or even gal-1 or gal-9 administration [186].

### 3.9. Serotonin and Receptors

Serotonin, or 5-hydroxytryptamine (5-HT), was initially discovered as a part of the coagulation process [195] and was later described in the homeostasis of several tissues such as the CNS [196] and the intestine [197, 198]. Enterochromaffin cells (ECs) of the intestinal mucosa synthesize, store, and release most of the serotonin in the body near the terminal of intestinal nerves [199, 200]. Other intestinal components such as enteric neurons and myenteric plexus cells also produce serotonin [201]. In addition, immune cells, enterocytes, and enteric neurons can express different subtypes of 5-HT receptors activated by 5-HT, which reveals the connection between the three systems: neuronal, immune, and endocrine [202]. Serotonin is known to be important in the modulation of the inflammatory process.

Changes in serotonin levels and metabolism receptors and intermediates were related to the progression of gastrointestinal diseases such as irritable bowel syndrome [203], IBD [204], celiac disease [205], and intestinal infectious diseases such as *Vibrio cholerae* [206, 207], *Salmonella typhimurium* [208], and *Trichinella spiralis* [209] infections. Changes in serotonin metabolism were shown to be related to the induction of inflammation and intestinal physiological changes [210].

Discordant evidence shows that the role of serotonin in CD depends on the progression of the digestive form. Some studies suggest that serotonin plays an anti-inflammatory role in the intestine of *T. cruzi-*infected patients without organ involvement, in which serotonin level is high and B and T lymphocyte [211] and MC [138] levels are low. The opposite was observed in megacolon, in which the amount of these inflammatory cells was higher than that of serotonin [138, 211]. In contrast, the serotonergic activity was greater in the intestinal crypt cells in Chagasic megacolon than in noninfected people and patients with idiopathic megacolon [32].

The anti-inflammatory function of serotonin was reinforced by showing that lymphocytes present in the intestine of infected patients without megacolon expressed greater amount of serotonin's 5-HT3A receptor than those in patients with megacolon [212]. In addition, CD8^+^ and CD20^+^ lymphocytes were higher in the most severe form than in the nondigestive form of CD [212]. These results corroborate the initial discussion on the immune suppressor role of serotonin [138, 211]. They also contribute to the development of a new pathway, in which this molecule would act on lymphocytes via the 5-HT3A receptor, leading to the inhibition of these cells. This would prevent the exacerbated immune response in CD patients without megacolon [212].

However, more information regarding the role of serotonin in the acute and chronic phases of CD is needed. The diversity of results found with either increased or decreased serotonin in Chagasic megacolon can be attributed to the methodological differences related to the phase of the disease, comparative groups, and experimental models used in each study.

A summary of biomarkers potentially involved in digestive CD establishment and/or progression is presented in Table 1.

## 4. Outstanding Questions for Further Scrutiny

The data discussed in this review raises the following questions to help clarify the several components involved in the intestinal form of CD:

- Does the activation of ECGs with a proinflammatory profile precede neural destruction in Chagasic megacolon? What is the behavior of ECGs in the acute phase of CD?
- Do ECGs influence intestinal motility and mucosal changes in Chagasic megacolon?
- Are ECGs protective or harmful in the progression of Chagasic megacolon?
- Would it be possible to stimulate neuronal regeneration in the intestine of CD patients with megacolon?
- Does serotonin have different effects in acute and chronic phases of *T. cruzi* infection? What is the relationship between serotonin and immune system activation or inhibition in Chagasic megacolon?
- Would MC enzymes, such as tryptase, be another neuronal cell death mechanism and maintaining the inflammatory perfil in Chagasic megacolon?
- What is the function of chymase in intestinal tissue remodeling in Chagasic megacolon?
- Would galectins be involved in the maintenance of the inflammatory process and intestinal remodeling in CD patients with megacolon?
- What is the role of galectins in the protection or intestinal neuronal damage in CD?

The answer to any of these questions may be related to the development of new therapies to prevent, improve, or even reverse the severity of Chagasic megacolon.

## 5. Conclusion

Although over 100 years have passed since the discovery of CD, little is known about the specific mechanism of Chagasic megacolon pathogenesis. This review shows that Chagasic patients with or without megacolon have a rich and differentiated intestinal microenvironment. Additionally, the participation of neurons, glial cells, MCs, eosinophils, and proteins addressed in this review reveals the high complexity of Chagasic megacolon pathology. The progression of the digestive form of *T. cruzi* infection is associated with different components that circulate and interact with the immunological, endocrine, and neurological systems. Glial cells and MCs are potential participants in its systems since they are sensitive to stimuli such as proinflammatory cytokines, serotonin, and galectins. Thus, a more profound analysis of these components could contribute to clinical management and the identification of new therapeutic targets.

## Figures and Tables

**Table 1:** Intestinal microenvironment and biological behavior of the components involved in the pathophysiology of Chagasic megacolon.

Biomarker or techniques	Markers	Behavior	Supposed participation in the pathogenesis	References
PGP 9.5	Pan-neural	↓	Destruction induced by the parasite and the inflammatory process established in the intestine. It affects intestinal motility and is involved with fecal stasis, organ dilation, and Chagasic megacolon progression.	[15, 34]
Peripherin	Pan-neural	↓		[50, 112]
	Disregarded	-		[48, 57]
HuC/HuDa	Pan-neural	↓		[56]
Substance P	Excitatory motor neurons (SP)	↑	Maintenance of the intestinal proinflammatory profile.	[56, 77]
GAP-43	Excitatory motor neuron regeneration (GAP-43/SP and GAP-43/cCHAT)	=	The greatest regeneration found in the subpopulations of inhibitory motor neurons (GAP-43/VIP and GAP-43/NO) is related to the greater destruction of these cell types when compared to the other subpopulations, which do not suffer reduction. Thus, through compensatory mechanisms, the subpopulation of inhibitory neurons tries to be reestablished, and due to this, there is only an increase in this subpopulation.	[50]
	Inhibitory motor neuron regeneration (GAP-43/VIP and GAP-43/NO)	↑		
	Intrinsic primary afferent neuron regeneration (GAP-43/calretinin)	=		
	Interneuron regeneration (GAP-43/neuropeptide Y)	=		
S-100	Enteric glial cells	↓	Infection focus in the intestine.	[15, 21, 114]
GFAP	Enteric activated glial cells	↑	Increased activation of this cell type in an attempt to control the infection through the production of proinflammatory and microbicidal components, which leads to neurotoxicity or/and increased activation of this cell type in an attempt to control the inflammatory process through the production of anti and regulatory cytokines and neuroprotective components.	[15, 21]
CD3^+^	T lymphocyte	↑	Participation of the immune response in neuronal loss.	[8, 15]
CD20^+^	B lymphocyte	↑		
CD68^+^	Macrophage	↑		[15]
CD57^+^	Natural killer cell	↑		
TIA-1+	Cytotoxic lymphocyte	↑		
Hematoxylin–eosin section	Eosinophil	↑	Participation of the immune response in neuronal loss and intestinal remodeling.	[13]
Giemsa/toluidine blue section	Mast cell	↑		[13, 139]
Serotonin	Serotonin-producing cells	↓	Duality between the anti-inflammatory and proinflammatory role of the components.	[138, 211]
		↑		[32]
5-HT3a receiver	Lymphocytes producing 5-HT3a receptor	↓		[212]
Tryptase	Tryptase-producing mast cells	↑	Participation in neuronal damage, maintenance of the inflammatory process, and tissue remodeling in the intestine.	[34, 158]
Chymase	Chymase-producing mast cells	↑		[34]
Galectin-1 and -9	Galectins	↑	Neuroprotection and infection control.	[182]
Galectin-3	Galectins	↑	Maintenance of the inflammatory process, neuroinflammation, and fibrosis.	[182, 183]

↑: increased component compared to healthy intestine without *T. cruzi* infection; ↓: decreased component compared to healthy intestine without *T. cruzi* infection; =: no component change compared to healthy intestine without *T. cruzi* infection.

## Data Availability

All the data used to support the findings of this study are included within the article and references.
